# Emergence and Spread of Cephalosporin-Resistant *Neisseria gonorrhoeae* with Mosaic *penA* Alleles, South Korea, 2012–2017

**DOI:** 10.3201/eid2503.181503

**Published:** 2019-03

**Authors:** Hyukmin Lee, Young Hee Suh, Sunhwa Lee, Yong-Kyun Kim, Mi-Soon Han, Hye Gyung Bae, Magnus Unemo, Kyungwon Lee

**Affiliations:** Yonsei University College of Medicine, Seoul, South Korea (H. Lee, Y.H. Suh, K. Lee);; Seegene Medical Foundation, Seoul (S. Lee);; Samkwang Medical Laboratories, Seoul (Y.-K. Kim); U2 Clinical Laboratories, Seoul (M.-S. Han);; Green Cross Laboratories, Yongin, South Korea (H.G. Bae);; World Health Organization Collaborating Centre for Gonorrhoea and other STIs, Örebro University, Örebro, Sweden (M. Unemo)

**Keywords:** Neisseria gonorrhoeae, bacteria, gonorrhea, antimicrobial treatment, antimicrobial resistance, ceftriaxone, cefixime, penA, NG-MAST, MLST, South Korea, cephalosporin, sexually transmitted infections, AMR

## Abstract

In South Korea, surveillance of antimicrobial drug resistance in *Neisseria gonorrhoeae* is extremely limited. We describe the emergence and subsequent national spread of *N. gonorrhoeae* strains with mosaic *penA* alleles associated with decreased susceptibility and resistance to extended-spectrum cephalosporins. From 2012 through 2017, the proportion of mosaic *penA* alleles in gonococcal*-*positive nucleic acid amplification test (NAAT) specimens across South Korea increased from 1.1% to 23.9%. Gonococcal strains with mosaic *penA* alleles emerged in the international hubs of Seoul in Gyeonggi Province and Busan in South Gyeongsang Province and subsequently spread across South Korea. Most common was mosaic *penA-*10.001 (n = 572 isolates; 94.7%), which is associated with cefixime resistance. We also identified mosaic *penA-*34.001 and *penA*-60.001, both of which are associated with multidrug-resistant gonococcal strains and spread of cefixime and ceftriaxone resistance. Implementation of molecular resistance prediction from *N. gonorrhoeae*–positive nucleic acid amplification test specimens is imperative in South Korea and internationally.

*Neisseria gonorrhoeae* remains a major public health threat globally (*1*). During the past 2 decades, *N. gonorrhoeae* strains with resistance to extended-spectrum cephalosporins (ESCs), including ceftriaxone, the last remaining option for empiric first-line gonorrhea treatment, have emerged and spread internationally, which is a serious concern worldwide (*2**–**23*). Enhanced global antimicrobial resistance (AMR) surveillance is crucial to controlling further spread of AMR strains. However, in many countries, culture-based AMR testing cannot be performed because most patients are treated empirically at primary-care facilities or hospitals where culture is unavailable and nucleic acid amplification tests (NAATs) have replaced culture for diagnosis. Consequently, molecular methods for detection of AMR determinants in *N. gonorrhoeae*–positive NAAT specimens are essential (*21*).

For the currently recommended ESCs, resistance is influenced by many different mutations in various genes that collaboratively increase the MICs of ESCs (*4*,*5*,*7*,*24*). Nevertheless, the main mechanism for ESC resistance is the acquisition of a mosaic *penA* allele encoding a mosaic version of the ESC lethal target, penicillin-binding protein 2 (*24**–**32*). Nearly all *N. gonorrhoeae* isolates with clinical or in vitro resistance to ESCs contain a mosaic *penA* allele. However, isolates with decreased susceptibility or susceptibility to ESCs also can contain a mosaic *penA* allele; that is, different mosaic *penA* alleles can affect the ESC MICs differently (*3**–**8*,*10**–**19*,*24**–**38*). Accordingly, a molecular assay detecting all types of mosaic *penA* alleles can predict ESC resistance with a high sensitivity but lower specificity (*24**–**31*). Currently, no molecular assays for detection of mosaic *penA* alleles and prediction of ESC resistance are commercially available.

In South Korea, ≈15,000 gonorrhea patients (≈29 patients/100,000 population) are reported to the Korean Health Insurance System (http://opendata.hira.or.kr) annually, but culture and AMR testing of *N. gonorrhoeae* are exceedingly limited (*2*,*3*). ESC resistance and ESC-resistant *N. gonorrhoeae* strains with the mosaic *penA*-10.001 allele were observed in 2011 in South Korea (*3*). Since the early 2000s, ESC-resistant *N. gonorrhoeae* strains containing the mosaic *penA*-10.001, associated with cefixime resistance, have been prevalent and caused cefixime treatment failures in Japan (*4*,*5*,*7*,*33*). In South Korea, molecular prediction of AMR directly from *N. gonorrhoeae*–positive NAAT samples is imperative for large scale screening and prediction of the level and spread of ESC resistance in Korea.

We describe the emergence and subsequent national spread of *N. gonorrhoeae* strains with mosaic *penA* alleles in gonococcal-positive NAAT specimens across South Korea from 2012 through 2017. For molecular epidemiology, *penA* sequencing, *N. gonorrhoeae* multiantigen sequence typing (NG-MAST), and multilocus sequence typing (MLST) were performed on all mosaic *penA*-positive specimens.

## Materials and Methods

### *N. gonorrhoeae*–Positive NAAT Specimens

We examined DNA extracts of NAAT specimens, mainly first-voided urine (55%) and vaginal swab specimens (27%), but also urethral and cervical swab specimens (18%), positive for *N. gonorrhoeae* (n = 3,884; 1 specimen per patient) by Seeplex NAATs (Seegene, http://www.seegene.com). These DNA extracts were obtained from commercial centralized accredited laboratories (2012–2017, Seegene Medical Foundation and SamKwang Medical Laboratories; 2016–2017, U2Bio; and 2017, Green Cross Medical Foundation) in Seoul, South Korea. The DNA extracts were stored at −70°C before further analysis. 

All samples were collected and preserved as part of the routine diagnostics, and no patient identification data were available during the study. Therefore, ethics approval was not required.

### Detection of Mosaic *penA* Alleles

We examined all DNA extracts for mosaic *penA* alleles in 2 steps: 1) screening of all types of mosaic *penA* alleles (*7*,*34*); and 2) detection of the specific mosaic *penA* alleles that caused high-level resistance to ceftriaxone in the strains H041 (*penA*-37.001; *7*) and F89 (*penA*-42.001; *6*). For screening of all types of mosaic *penA* alleles, we used a previously described TaqMan probe–based real-time PCR method on a Rotor-Gene 6000 (QIAGEN, https://www.qiagen.com) (*34*). We then tested all DNA extracts positive for a mosaic *penA* allele by using a real-time PCR specific for *penA*-37.001 (*35*) and a modified hybridization probe-based real-time PCR detecting *penA*-42.001 (*36*).

### Sequencing of Mosaic *penA* Alleles

We sequenced the entire *penA* gene in all DNA specimens positive for a mosaic *penA* allele, as previously described (*3*,*37*). Among 604 mosaic *penA*-positive specimens, we were able to sequence 601 (99.5%) specimens. Three (0.5%) specimens failed to sequence due to low DNA content. The mosaic *penA* alleles were named by using the *Neisseria gonorrhoeae* Sequence Typing for Antimicrobial Resistance (NG-STAR; *38*) database (https://ngstar.canada.ca/welcome/home).

### Molecular Epidemiologic Characterization

We performed NG-MAST (*39*) and MLST on all mosaic *penA-*positive specimens using the methods described on the NG-MAST (http://www.ng-mast.net) and MLST (http://pubmlst.org/neisseria) websites. We successfully typed 594 (98.3%) of the mosaic *penA*-positive specimens with NG-MAST and 593 (98.2%) specimens with MLST. 

### Antimicrobial Agents Used for Gonorrhea Treatment

We acquired prescription data for patients treated for gonorrhea from 2010 through 2017 in South Korea from the Korea Health Insurance Review & Assessment Service (http://opendata.hira.or.kr). In South Korea, all clinics and hospitals participate in the national health insurance system, and reporting of prescription data and diagnosis to the Korea Health Insurance Review & Assessment Service HIRA is mandatory. We analyzed the percentage of diagnosed gonococcal infections in patients who did not have additional sexually transmitted infections and who were treated with each specific antimicrobial drug.

### Statistical Analysis

We used the Student *t*‐test and a test of proportions for statistical analysis (Statistica 12.0 PL software, https://www.tibco.com). The level of significance was set at α = 0.05.

## Results

### *N. gonorrhoeae*–Positive NAAT Specimens and Corresponding Patients

The collection of DNA extracts we examined consisted of 3,884 *N. gonorrhoeae*−positive NAAT specimens collected across South Korea from 2012 through 2017 (Table 1). The number of specimens per year varied from 428 in 2012 to 901 in 2017 (Table 1). Data on the sex and age of patients were available for 3,422 (88.1%) of specimens. The ratio of men to women was 1:0.37, and 71.1% of the specimens were collected from patients in their 20s (43.9%) and 30s (27.2%).

**Table 1:** Number of *Neisseria gonorrhoeae*–positive nucleic acid amplification test specimens examined, by year and location, South Korea, 2012–2017

Location	No. (%) tested specimens						
2012	2013	2014	2015	2016	2017	Total
Seoul metropolitan area	192 (44.9)	171 (32.6)	185 (30.6)	141 (23.3)	338 (41.2)	252 (28.0)	1,279 (32.9)
Gyeonggi area, including Incheon	71 (16.6)	96 (18.3)	100 (16.6)	156 (25.7)	191 (23.3)	157 (17.4)	771 (19.9)
Gangwon	4 (0.9)	12 (2.3)	7 (1.2)	19 (3.1)	16 (1.9)	14 (1.6)	72 (1.9)
North Chungcheong	6 (1.4)	1 (0.2)	16 (2.6)	19 (3.1)	25 (3.0)	73 (8.1)	140 (3.6)
South Chungcheong, including Daejeon	22 (5.1)	21 (4.0)	69 (11.4)	62 (10.2)	53 (6.5)	103 (11.4)	330 (8.5)
North Jeolla	2 (0.5)	2 (0.4)	13 (2.2)	12 (2.0)	24 (2.9)	42 (4.7)	95 (2.4)
South Jeolla, including Gwanju	17 (4.0)	6 (1.1)	39 (6.5)	43 (7.1)	42 (5.1)	61 (6.8)	208 (5.4)
North Gyeongsang, including Daegu	49 (11.4)	93 (17.7)	78 (12.9)	93 (15.3)	83 (10.1)	103 (11.4)	499 (12.8)
South Gyeongsang, including Busan and Ulsan	65 (15.2)	121 (23.1)	97 (16.1)	61 (10.1)	46 (5.6)	93 (10.3)	483 (12.4)
Jeju	0	1 (0.2)	0	0	3 (0.4)	3 (0.3)	7 (0.2)
Total	428 (100)	524 (100)	604 (100)	606 (100)	821 (100)	901 (100)	3,884 (100)

### Molecular Typing of Gonococcal Mosaic *penA*-Positive Specimens

The proportion of mosaic *penA* alleles in the specimens increased annually, from 1.2% (5/428) in 2012 to 23.9% (215/901) in 2017 (Table 2). The mosaic *penA*-10.001, previously associated with resistance to ESCs, particularly cefixime and other oral ESCs (*4*,*5*,*33*), was the most common mosaic *penA* allele (n = 572; 94.7% of all mosaic *penA-*positive specimens). The annual proportion of mosaic *penA*-10.001 among the mosaic *penA*-positive specimens varied from 60% to 100% (Table 2). Most (76.9%) mosaic *penA*-10.001−positive samples belonged to MLST ST1901, but the proportion of sequence type (ST) 1901 among the mosaic *penA*-10.001 specimens decreased from 100% (5/5) in 2012 to 68% (143/210) in 2017. In contrast, the proportion of other MLST STs, including ST1588 and ST7363, increased. In 2012 and 2013, NG-MAST ST2958 was the most prevalent ST, but the genetically closely related NG-MAST ST10668 was most common in 2014 and 2015. In 2016 and 2017, NG-MAST STs diversified, and ST15014 became the most common. 

**Table 2:** Molecular typing of specimens positive for a *Neisseria gonorrhoeae* mosaic *penA* allele by year, South Korea, 2012–2017*

Year	No. (%) specimens with mosaic *penA*	Mosaic *penA* allele (no. specimens)	MLST ST (no. specimens)	NG-MAST ST (no. specimens)
2012	5 (1.2)	10.001 (5)	1901 (5)	2958 (5)
2013	27 (5.2)	10.001 (17)	1901 (21)	2958 (8), 10668 (6), 5682 (1), 6783 (1), 10669 (1)
		72.001 (4)		1407 (3), 436 (1)
		10.001 (1)	7363 (1)	5308 (1)
		10.001 (3)	ND (5)	ND (3)
		34.001 (2)		10670 (1), ND (1)
2014	60 (9.9)	10.001 (1)	1588 (1)	6696 (1)
		10.001 (54)	1901 (56)	10668 (33), 2958 (15), 11495 (2), 11493 (1), 11494 (1), 11496 (1), 11497 (1)
		34.001 (1)		5622 (1)
		72.001 (1)		1407 (1)
		10.001 (3)	ND (3)	ND (3)
2015	109 (18.0)	10.001 (2)	1579 (2)	13376 (2)
		10.001 (7)	1588 (7)	12399 (3), 3611 (2), 6696 (1), 10679 (1)
		10.001 (82)	1901 (82)	10668 (38), 2958 (20), 6734 (7), 11495 (4), 1407 (3), 6481 (3), 4502 (2), 6373 (2), 11497 (1), 13374 (1), 13388 (1), 13391 (1)
		10.001 (1)	1922 (1)	13393 (1)
		10.001 (10)	7363 (10)	5308 (8), 11562 (1), 13389 (1)
		10.001 (2)	7371 (2)	13390 (1), 13394 (1)
		34.001 (2)	7827 (2)	3702 (2)
		10.001 (2)	8137 (2)	13373 (1), 13392 (1)
		10.001 (1)	10931 (1)	12402 (1)
2016	185 (22.5)	10.001 (11)	1588 (11)	15024 (7), 15026 (3), 15086 (1)
		10.001 (133)	1901 (148)	15014 (39), 15016 (31), 15015 (28), 7307 (8), 15025 (3), 15041 (3), 15029 (2), 15045 (2), 11497 (1), 15013 (1), 15017 (1), 15018 (1), 15019 (1), 15020 (1), 15021 (1), 15022 (1), 15023 (1), 15027 (1), 15031 (1), 15033 (1), 15034 (1), 15038 (1), 15042 (1), 15044 (1), 15048 (1)
		27.001 (2)		15015 (2)
		34.001 (3)		4431 (1), 15032 (1), 15039 (1)
		72.001 (10)		15018 (7), 4431 (1), 15016 (1), 15030 (1)
		10.001 (26)	7363 (26)	6910 (13), 15028 (4), 15037 (2), 15046 (2), 15012 (1), 15040 (1), 15043 (1), 15049 (1), 15050 (1)
2017	215 (23.9)	10.001 (40)	1588 (40)	15024 (24), 5576 (4), 3611 (3), 15025 (3), 15026 (2), 6696 (1), 7684 (1), 14668 (1), 16331 (1)
		10.001 (143)	1901 (145)	15014 (33), 15016 (25), 7307 (17), 15015 (16), 5446 (10), 15032 (3), 16324 (3), 16341 (3), 6734 (2), 15045 (2), 16270 (2), 16325 (2), 16335 (2), 12402 (1), 13973 (1), 15018 (1), 15019 (1), 15028 (1), 15029 (1), 16319 (1), 16320 (1), 16321 (1), 16323 (1), 16328 (1), 16329 (1), 16332 (1), 16333 (1), 16334 (1), 16336 (1), 16338 (1), 16342 (1), 16343 (1), 16344 (1), 16346 (1)
		34.001 (2)		15032 (2)
		10.001 (1)	1902 (1)	16339 (1)
		60.001 (1)	1903 (1)	16327 (1)
		10.001 (24)	7363 (25)	6910 (13), 16322 (3), 13044 (1), 13876 (1), 15037 (1), 15046 (1), 16318 (1), 16337 (1), 16340 (1)
		34.001 (1)		11624 (1)
		10.001 (1)	7371 (1)	16330 (1)
		10.001 (1)	10899 (1)	11895 (1)
		10.001 (1)	11179 (1)	16345 (1)

*MLST, multilocus sequence typing; ND, not determined (due to insufficient amount of DNA); NG-MAST, *N. gonorrhoeae* multiantigen sequence typing; ST, sequence type.

The mosaic *penA*-34.001 was found in 1–3 samples annually between 2013 and 2017, and the mosaic *penA*-72.001 (*penA*-34.001+P551S) was identified in 10 samples between 2013 and 2016. These mosaic *penA* alleles also have been previously associated with ESC resistance internationally (*4*,*5*,*31*,*40*). Of particular concern, mosaic *penA*-60.001, which has been found in internationally spreading ceftriaxone-resistant gonococcal strains since 2015 (*12*,*14**–**16*,*18*,*19*), was identified in a sample in 2017. This sample belonged to MLST ST1903 and NG-MAST ST16327 and was collected from a man in Seoul (Table 2).

### Distribution of Mosaic *penA* Alleles in Different Provinces 

**Figure 1:**
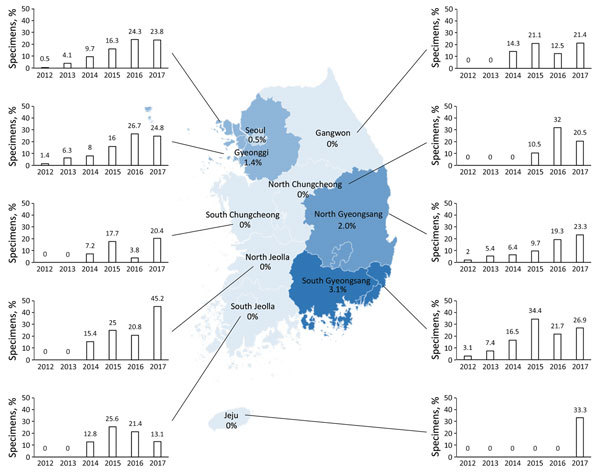
Distribution of specimens positive for a *Neisseria gonorrhoeae* mosaic *penA* allele, by year and province, South Korea, 2010–2017. Numbers shown in each province denote the proportion of samples positive for a mosaic *penA* allele in 2012. The bar graphs describe the percentage of specimens positive for a *N. gonorrhoeae* mosaic *penA* allele in each province and year. Seoul is the capital city of South Korea, and Gyeonggi Province contains an international airport.

We summarized the annual proportions of mosaic *penA* alleles in the 10 different provinces of South Korea from 2012 through 2017 (Figure 1). In 2012, only 5 mosaic *penA*-positive specimens were identified: 1 each in the capital city Seoul (0.5% of the total number of isolates collected in this location), Gyeonggi Province surrounding Seoul (1.4%), and North Gyeongsang Province (2.0%) and 2 (3.1%) in South Gyeongsang Province, which includes Busan, the second largest city in South Korea. In 2013, the proportions of mosaic *penA*-positive specimens in these 4 provinces increased to 4.1%–7.4%, but no mosaic *penA*-positive specimens were detected in any of the other provinces. However, in 2014, while the proportion of mosaic *penA*-positive specimens further increased in these 4 provinces (to 6.4%–16.5%), mosaic *penA*-positive specimens also emerged in 4 of the 6 additional provinces (proportions of 7.2%–15.4%). Mosaic *penA*-positive specimens were widely spread in all provinces of South Korea, except Jeju Island, in 2015 (9.7%–34.5%) and 2016 (3.8%–32.0%). In 2017, the proportion of mosaic *penA*-positive specimens was >20% in all provinces, except South Jeolla Province (13.1%), and mosaic *penA*-positive specimens also were found on Jeju Island (33.3%) (Figure 1).

### Antimicrobial Drugs Used for Gonorrhea Treatment 

**Figure 2:**
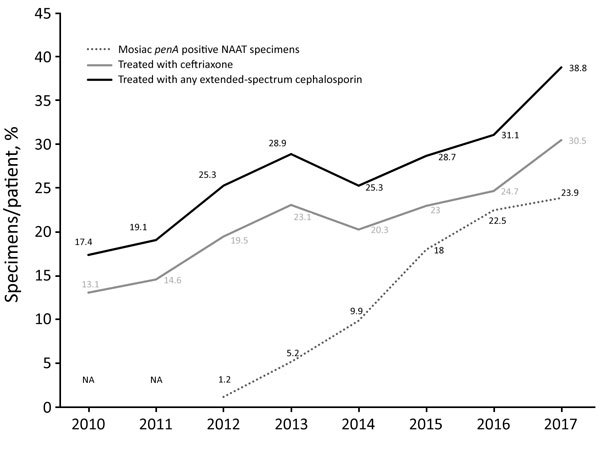
Percentages of *Neisseria gonorrhoeae* mosaic *penA* in NAAT specimens and gonorrhea patients treated with ceftriaxone or any extended-spectrum cephalosporin, South Korea, 2010–2017. Data for antimicrobial drug use were acquired from the Korea Health Insurance Review and Assessment Service in South Korea. In 2010 and 2011, no NAAT samples were available for screening of mosaic *penA* alleles. NA, not assessed; NAAT, nucleic acid amplification test.

In 2010, 13.1% of gonorrhea patients were treated with ceftriaxone, and 17.4% were treated with any ESC, but these proportions increased to 30.5% treated with ceftriaxone and 38.8% treated with ESCs in 2017 (Figure 2). With the exception of ceftriaxone, cefixime is the most widely used ESC; the proportion of patients treated with cefixime increased from 2.9% in 2010 to 6.6% in 2017. In 2010, 32.8% of patients were treated with spectinomycin and 49.6% with fluoroquinolone, but these proportions decreased in 2017 to 16.0% treated with spectinomycin and 26.7% treated with fluoroquinolone (Table 3).

**Table 3:** Percentage of patients treated with each antimicrobial drug used against gonorrhea in South Korea, 2010–2017

Year	% Patients					
	Fluoroquinolones*	Spectinomycin	Ceftriaxone	Cefixime	Other cephalosporin	Penicillin G
2010	49.6	32.8	13.1	2.9	1.4	0.2
2011	51.4	29.2	14.6	3.7	0.8	0.2
2012	52.3	22.2	19.5	4.6	1.2	0.2
2013	47.0	23.9	23.1	4.6	1.3	0.2
2014	38.8	22.0	20.3	3.9	1.1	0.1
2015	39.3	17.7	23.0	4.5	1.1	0.1
2016	36.7	17.3	24.7	5.0	1.4	0.1
2017	26.7	16.0	30.5	6.6	1.7	0.1

*Mainly ciprofloxacin.

## Discussion

The emergence of multidrug-resistant *N. gonorrhoeae* is compromising the treatment of gonorrhea globally. In most countries, only ESCs, azithromycin combined with ESCs, and spectinomycin remain recommended for the empiric treatment of gonorrhea (*3**–**5*,*8**–**10*,*20**–**23*,*31*,*32*,*41**–**44*).

Among the ESCs, oral cefixime and the more potent injectable ceftriaxone have been recommended for the treatment of gonorrhea internationally (*4*,*5*,*10*,*41**–**44*). However, resistance to cefixime emerged in Japan in the early 2000s, and cefixime resistance subsequently has been reported in many countries globally. Since 2010, resistance to ceftriaxone, the last remaining option for empiric first-line monotherapy, also has been described in many countries (*3*–*19*,*21*,*32*). The mechanisms of resistance to these ESCs are complex and involve different mutations in the *penA* gene, in the promoter or coding region of *mtrR*, and in the *porB1b* gene. However, the main ESC-resistance determinant is evidently the acquisition of a mosaic *penA* allele. The mosaic *penA*-10.001 was shown to be strongly associated with cefixime resistance in gonococcal isolates in Japan in the early 2000s (*3**–**5*,*7*,*33*), which resulted in the exclusion of cefixime from the Japanese treatment guidelines in 2006 (*43*). In South Korea, 1 cefixime-resistant isolate cultured in 2004 (*2*), 1 in 2011, and 3 in 2013 previously were reported to contain mosaic *penA*-10.001 (*3*). In our study, 1.1% (n = 5) of *N. gonorrhoeae–*positive NAAT specimens contained a mosaic *penA* allele in 2012. This proportion increased significantly in 2017 to 23.9% (n = 215; p<0.05). The proportion of mosaic *penA-*positive specimens (n = 209) containing mosaic *penA*-10.001 was 100% (n = 5) in 2012 and >90% during all the following years, except 2013 (77.8%; 21/27).

Twenty-five (96.2%) of the NAAT specimens containing mosaic *penA*-10.001 in 2012 and 2013 were collected from the capital region of Seoul and surrounding Gyeonggi Province, as well as the Gyeongsang area (North and South). Seoul (population 8.8 million; 19.8% of the population of South Korea) and the Gyeonggi area (29.6% of the population) share 2 large international airports and an international port, which have pivotal roles in trading with foreign countries, including China and Japan. The South Gyeongsang area (15.6% of the population) includes Ulsan, Gyeonsangnam-do, and the second largest city in South Korea, Busan, which comprises the main port in northeast Asia, with close and frequent trading routes to Japan. In 2012 and 2013, the proportions of mosaic *penA*-10.001 were 2.2% in the capital area and 4.1% in the Gyeongsang area, whereas the proportion was only 0.8% in the remaining provinces. This indicates that *N. gonorrhoeae* strains with mosaic *penA*-10.001, associated with decreased susceptibility and resistance to ESCs, initially emerged or were imported to at least 2 areas (Seoul and Gyeongsang) of South Korea, possibly from Japan, where these strains have been prevalent for many years (*4*,*5*,*7*,*33*). From 2014, the proportion of *N. gonorrhoeae* mosaic *penA*-10.001 in the other provinces significantly increased (from 9.1% in 2014 to 20.9% in 2017; p<0.05); accordingly, *N. gonorrhoeae* strains with mosaic *penA*-10.001 spread and diversified, based on NG-MAST STs, nationally in South Korea.

Gonococcal resistance to cefixime and other oral ESCs is common in South Korea, according to a smaller study (*3*), and the prevalent mosaic *penA*-10.001 we report causes decreased susceptibility and frequently resistance to cefixime and other oral ESCs (*4*,*5*,*7*,*33*). With these facts in mind, oral ESCs should be abandoned from the treatment of gonorrhea in South Korea. Gonococcal strains with mosaic *penA*-10.001 also can cause ceftriaxone treatment failure (*17*). However, mosaic *penA*-10.001 affects the MICs of ceftriaxone less than those of cefixime in general. Thus, ceftriaxone, combined with azithromycin, should be the first-line empiric treatment in South Korea. However, *N. gonorrhoeae* specimens containing mosaic *penA*-34.001 (n = 11) and *penA*-72.001 (n = 15) also were detected. These mosaic *penA* alleles have been associated with ESC resistance globally (*4*,*5*,*31*,*40*) and require only a single additional penicillin binding protein 2 amino acid alteration (A501P) to develop high-level ceftriaxone resistance (*6*,*45*). Of most concern, mosaic *penA*-60.001 was found in a sample collected in Seoul in 2017 and this mosaic *penA* allele has been found in internationally spreading ceftriaxone-resistant gonococcal strains in Japan, Australia, Canada, Denmark, France, the United Kingdom, and Ireland since 2015 (*12*,*14**–**16*,*18*,*19*,*46*).

The detailed reasons for the emergence and subsequent national spread of gonococcal strains with mosaic *penA-*10.001 in South Korea are not clear. One possibility is importation of cefixime-resistant gonococcal strains containing the mosaic *penA*-10.001 from Japan, as previously mentioned. However, increased therapeutic use of ESCs, particularly oral ESCs, in South Korea cannot be excluded as contributing to the increased spread of ESC-resistant gonococcal strains. In South Korea, spectinomycin, fluoroquinolones, and ESCs have been used to treat gonorrhea since the early 2000s (*3*). In 2002, most patients were treated with spectinomycin; only 8.4% of all patients were treated with ceftriaxone, and oral ESCs were rarely prescribed. However, 38.8% of patients were treated with ESCs (30.5% with ceftriaxone) in 2017. Despite the high use of spectinomycin in South Korea for decades, susceptibility to spectinomycin in *N. gonorrhoeae* has remained high. No spectinomycin-resistant gonococcal strain has been reported since 1993. Nevertheless, spectinomycin has a low eradication rate for pharyngeal gonorrhea (*47*) and, if pharyngeal infection has not been excluded, should be used only in dual antimicrobial therapy (e.g., in combination with azithromycin). In the 2016 South Korea guideline, dual antimicrobial therapy (ceftriaxone 500 mg or 1 g plus azithromycin 1 g) is the recommended first-line empiric therapy for uncomplicated gonorrhea (*48*).

The limitations of our study include the unavailability of gonococcal isolates, MIC data, rectal or pharyngeal specimens (which are not covered by the national insurance system in South Korea), and clinical or epidemiologic information regarding patients or treatment outcome. Accordingly, phenotypic ESC resistance was not measured; instead molecular detection of mosaic *penA* alleles was used to reflect the ESC resistance. Nearly all gonococcal strains with ESC resistance contain a mosaic *penA* allele; however, isolates with decreased susceptibility or susceptibility to ESCs also can contain a mosaic *penA* allele, and different mosaic *penA* alleles affect the ESC MICs differently (*3**–**8*,*10**–**19*,*24**–**38*). Even so, the mosaic *penA*-10.001 in 94.7% of our mosaic *penA* allele-positive specimens is relatively strongly associated with resistance to cefixime particularly and other oral ESCs, although with a weaker association with ceftriaxone resistance. In addition, our molecular approach to identifying decreased susceptibility and resistance to ESCs can overestimate the ESC resistance. The mosaic *penA* allele PCR assay (*34*) did not include any internal control for gonococcal DNA. However, the assay has been previously evaluated on gonococcal strains and detects 1–10 copies of a mosaic *penA* per PCR reaction (*34*) on urogenital and extragenital gonococcal-positive and gonococcal-negative NAAT samples (*49*,*50*). Finally, the distribution of specimens could not be allocated by population in an ideal way and the suboptimal number of samples, especially from the Gyeonggi area, Jeju, Gangwon, South Gyeongsang, South Jeolla, and North Jeolla provinces in some years, might have underestimated the prevalence of *N. gonorrhoeae* specimens with mosaic *penA* allele in these provinces.

In conclusion, we describe the initial emergence and subsequent national spread of *N. gonorrhoeae* strains with mosaic *penA* alleles (*penA*-10.001 in 94.7% of specimens), associated with decreased susceptibility and resistance to ESCs, in NAAT specimens collected across South Korea from 2012 through 2017. The proportion of *penA*-10.001, frequently causing resistance to cefixime and other oral ESCs, increased during these years. Only a few additional mutations in *penA*-10.001 are required for development of high-level resistance to ceftriaxone. Furthermore, we identified mosaic *penA*-34.001 and *penA*-60.001, both associated with multidrug-resistant gonorrhea and international spread of cefixime and ceftriaxone resistance (*4*,*5*,*12*,*14**–**16*,*18*,*19*,*31*,*40*). The mosaic *penA*-60.001 has been found in internationally spreading ceftriaxone-resistant gonococcal strains in Japan, Australia, Canada, Denmark, the United Kingdom, France, and Ireland since 2015 (*12*,*14**–**16*,*18*,*19*,*46*). Nevertheless, *N. gonorrhoeae* strains with ESC resistance due to nonmosaic *penA* alleles, such as *penA*-13.001, also are spreading in South Korea (*3*). Consequently, it is essential to establish a systematic, regular, and quality-assured phenotypic AMR surveillance system for *N. gonorrhoeae* in South Korea. Implementing the use of molecular methods for prediction of AMR or antimicrobial susceptibility is also crucial. These molecular methods will effectively support phenotypic AMR surveillance and enable large-scale screening of *N. gonorrhoeae*–positive NAAT specimens, which represent most of *N. gonorrhoeae* diagnostic specimens in many countries internationally.
